# Structural Analysis of Porcine Reproductive and Respiratory Syndrome Virus Non-structural Protein 7α (NSP7α) and Identification of Its Interaction with NSP9

**DOI:** 10.3389/fmicb.2017.00853

**Published:** 2017-05-11

**Authors:** Jiaping Chen, Xiaodong Xu, Hu Tao, Yuan Li, Hao Nan, Yuanyuan Wang, Mengmeng Tian, Hongying Chen

**Affiliations:** ^1^College of Life Sciences, Northwest A&F UniversityYangling, China; ^2^College of Science, Northwest A&F UniversityYangling, China

**Keywords:** porcine reproductive and respiratory syndrome virus, non-structural protein 7α, protein structure, NMR, protein–protein interaction

## Abstract

Non-structural protein 7 (NSP7), which can be further cleaved into NSP7α and NSP7β, is one of the most conserved proteins of porcine reproductive and respiratory syndrome virus (PRRSV). NSP7 plays a role in provoking the humoral immune system in PRRSV-infected swine, but its structure and function are still not fully understood. Here, we analyzed the expression of NSP7, NSP7α, and NSP7β in PRRSV-infected MARC-145 cells. The solution structure of NSP7α was determined by using nuclear magnetic resonance (NMR). Although the structure provided little clue to its function, based on the structure of NSP7α, we predicted and further identified some key amino acids on NSP7α for the interaction of NSP7α with NSP9, the RNA dependent RNA polymerase of PRRSV. This study provided some new insights into the structure and function of PRRSV NSP7.

## Introduction

Porcine reproductive and respiratory syndrome virus (PRRSV) is an enveloped virus containing a positive single-stranded RNA genome of 15.4 kb. Together with lactate dehydrogenase elevating virus (LDV), equine arteritis virus (EAV), simian hemorrhagic fever virus (SHFV), and wobbly possum disease virus (WPDV), they form the family *Arteriviridae* (Plagemann and Moennig, 1992; Snijder and Meulenbdrg, 1998; Dunowska et al., 2012). PRRSV is the causative agent of PRRS, which is one of the most destructive disease in swine industry in the world. Based on genetic differences, PRRSV strains are divided into European genotype and North American genotype and the nucleotide identity between the two genotypes is only about 60% (Allende et al., 1999; Nelsen et al., 1999; Forsberg, 2005).

The genome of PRRSV contains at least nine open reading frames. Of these, ORF1a and ORF1b occupy 75% of the genome and encode polyproteins pp1a and pp1ab. Thereafter, the two polyproteins could be cleaved into non-structural proteins (NSP1-12) by proteases NSP1α, NSP1β, NSP2, and NSP4 (van Aken et al., 2006; Fang and Snijder, 2010; Li et al., 2015).

Among these NSPs, the roles of NSP5, NSP6, NSP7, and NSP12 in virus biology are still not understood.

ELISA analysis for NSP1, NSP2, NSP4, NSP7, and NSP8 showed NSP1, NSP2, and NSP7 could strongly react with pig serum, and the NSP7 dual ELISA can be used as a differential test for PRRSV serology (Brown et al., 2009). Previous research indicated that NSP3-8 might play a main role in PRRSV virulence (Kwon et al., 2008). A recent report showed that deletions in NSP7 led to failures in the recovery of PRRS virus (Zhang et al., 2013). These data suggest that the protein plays an important role in the life cycle of PRRSV.

With an internal cleavage site, NSP7 can be further cleaved into NSP7α and NSP7β (Li et al., 2012). The cleavage site located within NSP7 is conserved in arteriviruses and critical for the replication of EAV (van Aken et al., 2006). Nuclear magnetic resonance (NMR) assay of the structure of EAV NSP7α showed that the protein has three α-helices and five β-strands. Although the structure analysis possesses no recognizable functional motifs, EAV NSP7/NSP7α was proved to play an important role in viral RNA synthesis by structure-based reverse genetics studies (Manolaridis et al., 2011), consisting with the data from PRRSV (Zhang et al., 2013).

In this study, we analyzed the expression of NSP7α and NSP7β in PRRSV infected cells and determined the structure of NSP7α by NMR. Furthermore, we demonstrated the interaction of NSP7α with NSP9, the viral RNA dependent RNA polymerase (RdRp), and identified the key amino acids of NSP7α involved in NSP7α–NSP9 interaction by mutagenesis analysis.

## Materials and Methods

### Cells, Viral Strains

MARC-145 cells were maintained in Dulbecco’s modified Eagle’s medium (HyClone) supplemented with 10% fetal bovine serum (HyClone) at 37°C with 5% CO_2_. TA-12 (GenBank accession no. HQ416720.1), a highly pathogenic PRRSV isolate, was used in virus infection studies.

### Bioinformatics Analysis

The web tool of PDBeMotif^1^ was used for analyzing the motifs which may be functional in NSP7α. The alignment of the protein sequences were completed using DNAMAN software. PRISM2.0^2^ was used for the prediction of bonding sites between NSP7α and NSP9 (Tuncbag et al., 2011).

### Plasmid Constructs for the Expression of HIS-TEV-NSP7α/β

The full-length PRRSV cDNA clone FL12 of NVSL 97-7895 (GenBank accession no. AY545985.1) was used as the templates for the amplification of the cDNAs encoding NSP7α and NSP7β. The fragment of *nsp7*α or *nsp7*β was then inserted into a vector as described in a previous study (Chen et al., 2016), for the expression of NSP7α or NSP7β with an N-terminal HIS-tag and a TEV protease cleavage site between the tag and the fused protein.

### Polyclonal Antibodies against NSP7α and NSP7β

HIS-TEV-NSP7α and HIS-TEV-NSP7β were expressed in *E. coli* BL21 (DE3), and purified using Ni-NTA column (CWBIO, China). The 200 μg of each purified protein was mixed with equal volume of Freund’s complete adjuvant, and injected into rabbits in primary immunization. Rabbits were boosted three times at 2 weeks intervals, with mixture of 200 μg antigen in Freund’s incomplete adjuvant. The rabbit antiserums were collected 2 weeks after the last injection.

### Western Blot Assay

MARC-145 cells were infected with a MOI of 0.1, and lysed in SDS loading buffer at 8, 16, 24, 48, 72, and 96 h post infection (hpi). The proteins in cell lysis solution were separated by SDS-PAGE, and transferred onto a PVDF membrane. After blocking with 5% skimmed milk overnight at 4°C, the membranes were, respectively, incubated with anti-NSP7α and anti-NSP7β polyclonal antibodies, followed by incubation with goat-anti-rabbit IgG (CWBIO, China) conjugated horseradish peroxidase as secondary antibody. Immunodetection was performed using enhanced chemiluminescence (ECL) reagents (CWBIO, China) according to the supplier’s instruction.

### Immunofluorescence Assay

Porcine reproductive and respiratory syndrome virus-infected MARC-145 cells were fixed at 8, 16, 24, 48, and 72 hpi using 4% paraformaldehyde, permeabilized using 0.2% Triton X-100 for 10 min at room temperature, then blocked with 5% skimmed milk overnight at 4°C. Subsequently, the cells were, respectively, incubated with polyclonal antibodies against NSP7α and NSP7β for 1 h. After incubated with FITC-conjugated goat-anti-rabbit IgG (CWBIO, China) for 1 h, the cells were stained with PI for 5 min. The fluorescence images were captured using a confocal microscope (Nikon, Japan).

### Preparation of the NSP7α Solution for NMR Experiments

Uniformly labeled NSP7α was expressed in *E. coli* BL21 (DE3) grown in M9 minimal media containing 0.2% (w/v) ^13^C Glucose and 0.1% (w/v) ^15^N NH_4_Cl as the only carbon and nitrogen sources. HIS-tagged NSP7α was purified by affinity chromatography on a Ni-NTA column. The HIS-tag at the N terminus of NSP7α was removed overnight by TEV protease, subsequently. The final NMR sample was concentrated to about 0.8 mM in 20 mM KH_2_PO_4_ buffer (pH 7.5) with 92% H_2_O/8% D_2_O, 100 mM NaCl, and 1 mM TCEP. The residues of NSP7α were renumbered as residues 2–150 (the glycine from the C-terminus of TEV protease cleavage site was numbered as residue 1) in structural analysis.

### NMR Experiments, Analysis of Spectra, and Structure Calculation

NMR assignment of ^15^N, ^13^C labeled NSP7α was described in our previous report, and the ^1^H, ^13^C, and ^15^N chemical shifts for NSP7a were deposited in the BMRB under BMRB accession number 30014 (Chen et al., 2016). All the NMR experiments were carried out at 298K on a Bruker Avance 600 MHz spectrometer. All spectra were processed using NMRPipe/Draw (Delaglio et al., 1995) and analyzed using CARA^3^ (Keller, 2004).

The structure of NSP7α was refined by CNS (Brünger et al., 1998) based on the chemical shift assignments and NOE distance restraints. Backbone φ and ψ dihedral angle restraints obtained using TALOS+ (Shen et al., 2009). H-bond restraints were determined by secondary structure elements in initial structure. The final 20 structures with lowest energies were selected for representation and analyzed using PROCHECK_NMR (Laskowski et al., 1996). All representing structure figures were generated using PyMOL^4^.

### Yeast Two-Hybrid Analysis

To investigate the interaction of NSP7α with the other NSPs, yeast two-hybrid assays were performed. NSP7α cDNA fragment of NVSL 97-7895 was cloned into pGADT7 and the other NSPs’ cDNA fragments were inserted into pGBKT7. Primer sequences for *nsp7*α cloning were listed in **Table 1**. The pGBKT7 constructs were, respectively, co-transformed with pGADT7-NSP7α into *Saccharomyces cerevisiae* (yeast) strain Gold using the Yeastmaker Yeast Transformation System kit (Clontech, USA). Yeast co-transformed with pGBK7-P53 and pGADT7-T was used as a positive interaction control, and yeast co-transformed with pGBKT7-Lam and pGADT7-T was used as a negative control.

**Table 1:** The primers used in this study.

Primers	Sequences
EcoRINSP7α-F (yeast two-hybrid)	TAATGAATTCTCGCTGACTGGTGCCCTC
BamHINSP7α-R (yeast two-hybrid)	TAATGGATCCCTCCAGAACTTTCGGTGG
BamHINSP7α-F (pull-down assay)	TAATGGATCCTCGCTGACTGGTGCCCTC
XhoINSP7α-R (pull-down assay)	TAATCTCGAGTCACTCCAGAACTTTCGGT
NSP7αK61A-F	TTGATGCGGTTCGAGGTACTTTG
NSP7αK61A-R	TACCTCGAACCGCATCAACCTGCAC
NSP7αL69A-F	GGCCAAAGCTGAAGCTTTTGCTGAT
NSP7αL69A-R	AAGCTTCAGCTTTGGCCAAAGTACC
NSP7αF72A-F	CTTGAAGCTGCTGCTGATACCGTG
NSP7αF72A-R	ATCAGCAGCAGCTTCAAGTTTGGC

*Restriction enzyme sites are underlined.*

### Pull-Down Assay

The cDNA fragment encoding NSP7α was amplified by PCR using primer pairs BamHINSP7α-F/XhoINSP7α-R. The fragment of *nsp7*α was inserted into pTriEx-FLAG for the expression of FLAG-tagged NSP7α. The site-directed mutants of *nsp7*α were amplified using primers listed on **Table 1** and cloned into pTriEx-FLAG. NSP9 cDNA fragment was cloned into pTriEx1.1 vector to make pTriEx-NSP9 construct for the expression of HIS-tagged NSP9 (Liu et al., 2016).

Protein mixtures of FLAG-tagged NSP7α and HIS-tagged NSP9 were incubated for 1 h at room temperature. The solution was incubated with nickel magnetic beads (Millipore, USA) for 0.5 h at room temperature. After being washed with wash buffer (20 mM TRIS-HCl [pH 7.9], 300 mM NaCl, and 10 mM imidazole) for three times, the bound proteins were eluted with elution buffer (20 mM TRIS-HCl [pH 7.9], 300 mM NaCl, and 300 mM imidazole). Eluted proteins were separated by SDS-PAGE, and then detected by Western blot. FLAG-tagged and HIS-tagged proteins were, respectively, probed by mouse anti-FLAG antibody (CWBIO, China) and mouse anti-HIS (CWBIO, China) antibody, followed by incubation with goat-anti-mouse IgG (CWBIO, China) conjugated with horseradish peroxidase as secondary antibody. The protein mixture containing NSP7α and HIS-tagged TEV protease was used as negative control.

## Results

### Expression of NSP7α/β in the PRRSV-Infected Cells

**FIGURE 1:**
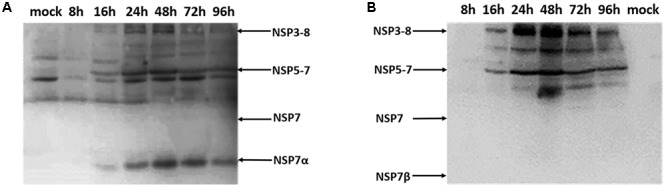
**Western blot analysis of the NSP7 expression in PRRSV-infected cells.** Cell lysates were collected at 8, 16, 24, 48, 72, and 96 hpi, and probed with antisera against NSP7α **(A)** and NSP7β **(B)**.

Antisera against NSP7α and NSP7β were used for the detection of NSP7α and NSP7β expression in PRRSV infected MARC-145 cells. By Western blot, anti-NSP7α antibodies detected a band of approximately 16 kDa, corresponding to the predicted size of NSP7α, by 16 hpi (**Figure 1A**). The production of this protein reached a peak level at 48 hpi, and the protein amount gradually decreased from 48 to 96 hpi. A series of protein bands at higher molecular weights were also recognized. These bands probably represented the polyprotein precursors of NSP7, and the products in accordance with the predicted size of NSP3-8 and NSP5-7 were marked with arrows in the figure. However, no protein with the predicted size of NSP7 were detected in the PRRSV infected cells.

The antiserum against NSP7β also recognized the polyprotein precursors NSP3-8 and NSP5-7 (**Figure 1B**). It detected neither NSP7 nor NSP7β in the PRRSV infected cell lysates, probably due to the instability characteristics of the proteins. These results were consisted with the previous study that was carried out using PRRSV monoclonal antibodies (Li et al., 2012).

**FIGURE 2:**
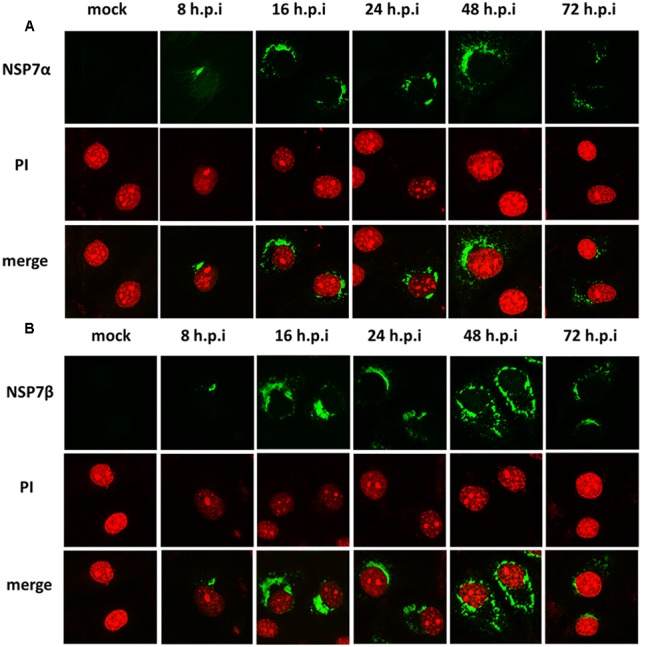
**Detection of NSP7α, NSP7β expression in PRRSV-infected cells.** MARC-145 cells were fixed at 8, 16, 24, 48, and 72 hpi and, respectively, stained with antisera against NSP7α **(A)** and NSP7β **(B)**. The fluorescence images were captured on a confocal microscope (Nikon, Japan).

To visualize the intracellular expression and localization of NSP7 and its precursors, PRRSV infected MARC-145 cells were stained with the antisera and observed by immunofluorescence microscopy. Although the antiserum against NSP7β did not detect the cleaved form of NSP7β in Western blot, the two sera gave similar results in immunofluorescence assay, probably because the antiserum against NSP7β could detect the NSP7 precursors as well as the antiserum against NSP7α. As shown in **Figure 2**, the signal for NSP7α and NSP7β could be detected in the infected cells as early as 8 hpi and the fluorescent foci were small and mostly concentrated on one side of the nucleus. After 16 hpi, the fluorescence signals for both NSP7α and NSP7β were more diffused in the cytoplasm of the infected cells. By 72 hpi, both the number of fluorescent cells and the intensity of the fluorescence obviously decreased. Protein degradation, and partially loss of the infected cells during the experimental process could both contribute to the reduction of the NSP proteins.

### Solution Structure of PRRSV NSP7α

**FIGURE 3:**
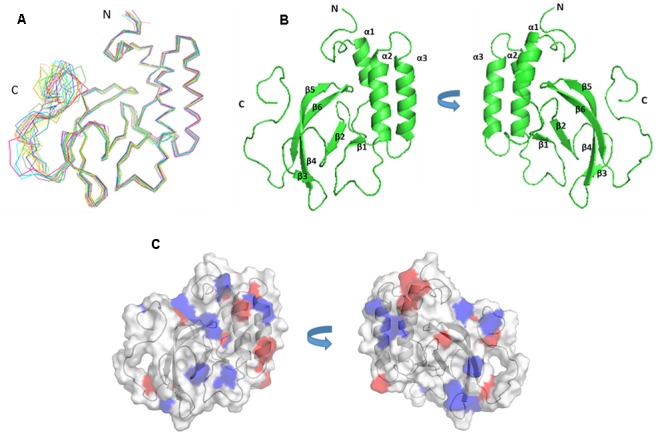
**The structure and surface features of PRRSV NSP7α determined by NMR Spectroscopy. (A)** Backbone trace of the ensemble of 20 superimposed lowest-energy structures of NSP7α in aqueous solution. **(B)** The cartoon representation of NSP7α structure in solution. **(C)** The surface representation of the solution structure of NSP7α. Negatively charged patches are colored in red, and positively charged regions are in blue.

The solution structure of PRRSV NSP7α was determined by NMR spectroscopy, and three dimensional structure was calculated using 2466 NOE distance restraints (inter-residue), 202 dihedral angle restraints and 85 H-bond restraints. Coordinates of the ensemble of the 20 lowest energy structures were deposited in the Protein Data Bank^5^ under PDB accession number 5I65. Parameters of the structure are listed in **Table 2**. As shown in **Figure 3A**, the ensemble of structures for the most domains are well superposed. NSP7α contains a bundle of three α-helices (α1 from residue Asp13 to Lys21, α2 from residue Phe41 to Gln56, α3 from residue Gln59 to Ala74) and six-strand mixed β-sheet (β1 containing residues Cys27 to Val29, β2 containing residues Ile86 to Leu90, β3 containing residues Phe99 to Val103, β4 containing residues His108 to Glu114, β5 containing residues Arg116 to Ala119 and β6 containing residues Met123 to Val129 in the order α1-β1-α2-α3-β2-β3-β4-β5-β6. The mixed β-sheet is formed by five antiparallel strands (β2 to β6) and one short parallel strand (β1) (**Figure 3B**).

**Table 2:** Summary of structural statistics for NSP7α structure.

Structural statistic	Value for NSP7α
Total distance restraints (inter-residue)	2466
Short range (residue i to i + j, j = 1)	938
Medium range (residue i to i + j, 2 ≤ j ≤ 4)	672
Long range (residue i to i + j, j > 4)	856
Hydrogen bonds	85
Total dihedral angle restraints	202
φ	101
ψ	101
Restraint violations	
Distance restraint violation 0.2 Å	0
Dihedral restraint violation 5°	0
Average rmsd (Å) among the 20 refined structures	
Backbone of structured regions (residues 3–129)	0.378 ± 0.102
Heavy atoms of structured regions (residues 3–129)	0.763 ± 0.079
Ramachandran statistics of 20 structures (% residues)	
Most favored regions	63.73
Additional allowed regions	33.33
Generously allowed regions	1.67
Disallowed regions	1.27

In order to search for the functional motifs of NSP7α, the protein structure was analyzed using web tools of PDBeMotif^6^, but no recognizable functional motifs was identified. The surface charge distribution of the protein shows that the protein has some scattered electropositive and electronegative patches, and more of the electropositive patches locate on the surface of the β-sheet (**Figure 3C**).

**FIGURE 4:**
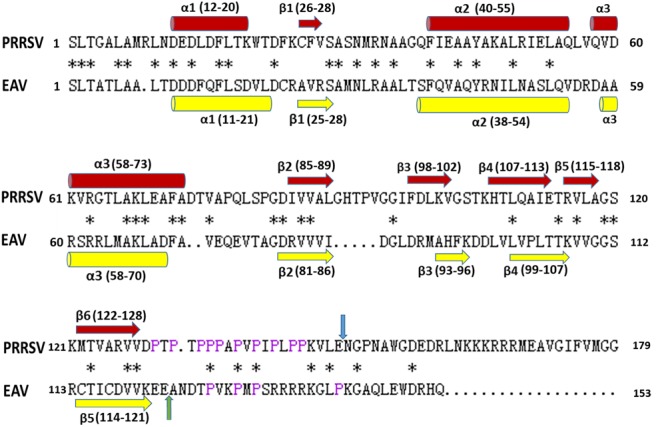
**Alignment of amino acid sequences of NSP7α.** PRRSV sequences are based on strain NVSL 97-7895 (GenBank accession no. AAS59260.1), and EAV sequences are from isolates Bucyrus (GenBank accession no. ABI64071.1). The conserved residues are marked with asterisks. The prolines at the C-terminus of PRRSV NSP7α and N-terminus of EAV NSP7β are colored in purple. The NSP7α/NSP7β cleavage sites of PRRSV and EAV are, respectively, indicated with blue arrow and green arrow. The α-helix and β-sheet regions are, respectively, marked with cylinders and arrows.

Sequence alignment shows that PRRSV (NVSL 97-7895) NSP7α shares 33.33% identity with EAV (Bucyrus strain) NSP7α (**Figure 4**). It is noteworthy that the C-terminus of PRRSV NSP7α has a predominant proline-rich region (PRR), with 10 prolines out of the last 20 amino acids. In comparison, the C-terminus of EAV NSP7α is shorter and lack the PRR, but its NSP7β N-terminus has four proline residues and five other same amino acids as the C-terminus of PRRSV NSP7α and shares less identity with the N-terminus of PRRSV NSP7β. Therefore, this discrepancy could be attributed to the position change of the cleavage site between NSP7α and NSP7β. It is unfortunate that the structure of the C-terminal PRR was not solved, as prolines lack the amide protons necessary for the standard triple resonance experiment and the backbone of this region was not able to be assigned.

**FIGURE 5:**
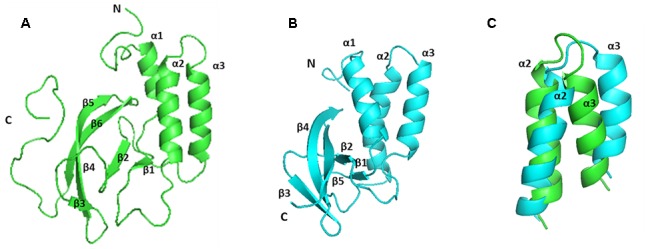
**Comparison of the solution structure of PRRSV and EAV NSP7α proteins. (A)** The structure of PRRSV NSP7α (PDB accession number: 5I65). **(B)** The structure of EAV NSP7α (PDB accession number: 2L8K). **(C)** Overlay of the region from helices α2 to α3. PRRSV NSP7α is colored in green and EAV NSP7α is in blue.

The solution structure of EAV NSP7α (PDB accession number: 2L8K) have previously been reported (Manolaridis et al., 2011). PRRSV NSP7α possesses similar secondary structures as EAV NSP7α (**Figures 4, 5A,B**). Both of them have three α-helices gathered together in the N-terminal half, and all the β-sheets of the two proteins are located at one side of the α-helices. Strand β4 and strand β5 of PRRSV NSP7α locate at a similar position with an equivalent length to the strand β4 of EAV NSP7α. However, overlay of the 3-D structure of the two proteins showed that the two structures were not well superimposed, and the best superimposed region located from helices α2 to helices α3 (**Figure 5C**).

### Interaction of NSP7α with NSP9

**FIGURE 6:**
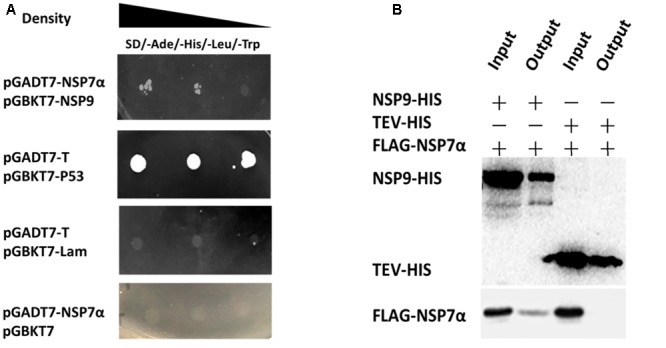
**Identification of the interaction of NSP7α with NSP9. (A)** Yeast two-hybrid assays. The yeast cells were co-transformed with pGADT7-nsp7α and pGBKT7-nsp9 constructs, subjected to 10-fold serial dilutions and plated on SD/-Ade/-His/-Leu/-Trp medium. The cells co-transformed with pGBK7-P53 and pGADT7-T were used as positive interaction controls, and the group with pGBKT7-Lam and pGADT7-T was used as a negative control. **(B)** Pull-down assays. FLAG-tagged NSP7α and HIS-tagged NSP9 were expressed in *E. coli*, mixed together, and the protein mixture was purified with nickel magnetic beads. HIS-tagged and FLAG-tagged proteins pulled down by the beads were, respectively, analyzed by Western blot with antibodies against HIS-tag and FLAG-tag.

Previous studies have reported that arterivirus NSP7α is essential for the viral replication and it plays an important role in viral RNA synthesis (Manolaridis et al., 2011; Zhang et al., 2013). It remains unknown whether the protein is involved in the replication and transcription complex (RTC) formation by interacting with other NSPs. To investigate the interaction of PRRSV NSP7α with other NSPs, yeast two-hybrid assays were carried out, and it was found that NSP7α could interact with NSP9 (**Figure 6A**), the viral RNA-dependent RNA polymerase. To validate the NSP7α–NSP9 interaction, FLAG-tagged NSP7α and HIS-tagged NSP9 were expressed in *E. coli* and used for pull-down assay. The data confirmed that NSP7α could bind to NSP9 *in vitro* (**Figure 6B**).

**FIGURE 7:**
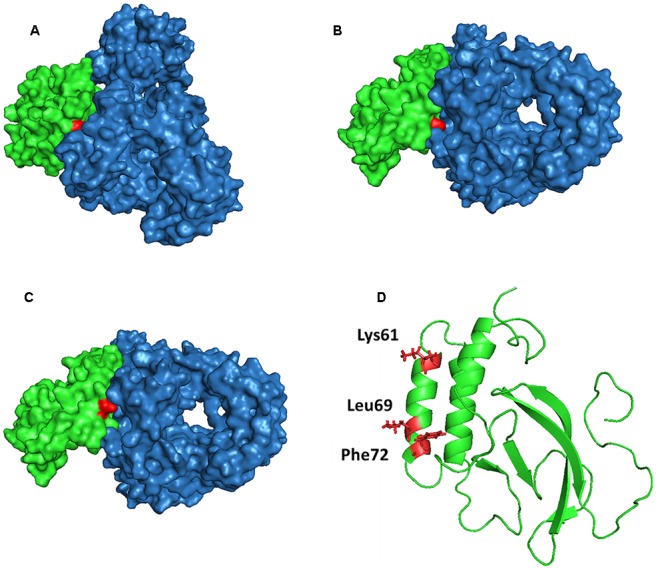
**Prediction model of NSP7α–NSP9 interaction (with evaluated energy of -27.86 kcal/mol).** The surface features of the NSP7α–NSP9 interaction model are shown in **(A–C)**. NSP7α is colored in green, NSP9 is in blue, and Lys61 **(A)**, Leu69 **(B)**, and Phe72 **(C)** are highlighted in red. **(D)** The three amino acid residues are highlighted in red in the cartoon representation of NSP7α structure.

**FIGURE 8:**
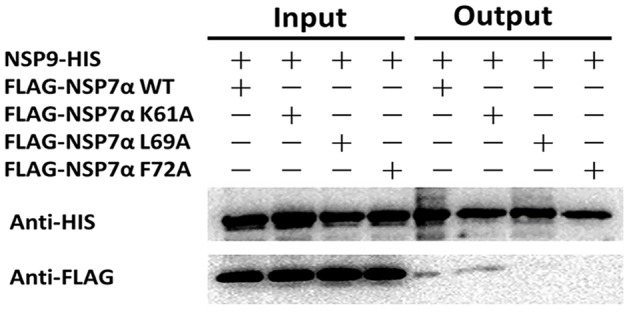
**Pull-down analysis of the interaction of NSP7α mutants with NSP9.** FLAG-tagged and HIS-tagged proteins were, respectively, detected by Western blot using antibodies against HIS and FLAG tag.

To investigate the interaction of NSP7α with NSP9 in more details, the 3-D structure of NSP9 was predicted (Liu et al., 2016), and used for the docking prediction of NSP7α–NSP9 interaction by using PRISM2.0 (Tuncbag et al., 2011). In the predicted interaction model with the lowest energy (-27.86 kcal/mol) among the outputs, three amino acids (K61, L69, and F72) in helix α3 of NSP7α were predicted to be involved in the recognition of NSP9 (**Figure 7**). Pull-down assay using the NSP7α mutants containing single substitutions at the three sites showed that mutations at L69 and F72 abolished the binding ability of NSP7α to NSP9, but the K61A mutant could interact with NSP9 as well as WT NSP7α (**Figure 8**). It is worth to note that L69 and F72 were conserved between PRRSV and EAV NSP7α, while K61 in PRRSV NSP7α was replaced by another basic amino acid R in EAV NSP7α (**Figure 4**). This result confirmed that helix α3 of NSP7α was a critical region involved in the NSP7α–NSP9 interaction, and it also indicated that the two conserved amino acids L69 and F72 were essential for the protein to maintain its ability binding to NSP9.

## Discussion and Conclusion

NSP7α is one of the most conservative NSPs of PRRSV and is an essential factor for the rescue of virus by reverse genetics (Zhang et al., 2013), but its structure and specific function in virus biology still remains unknown. To elucidate the structure and function of NSP7α, we analyzed its solution structure, the expression of the protein in PRRSV infected cells, and its interaction with NSP9 in this study.

Previous studies found that NSP7 of arterivirus EAV could be internally cleaved by NSP4, the viral protease, yielding small amounts of NSP7α and NSP7β. In infected cell lysates, NSP7 and its cleaved product NSP7α were detected by Western blot. Cleaved NSP7β was not detected, but mutagenesis analysis demonstrated that the internal processing of NSP7 was critical for virus reproduction (van Aken et al., 2006). A recent study on PRRSV NSP7 showed that only NSP7α was detectable in the infected cells, and neither NSP7 nor NSP7β were detected by Western blot and radioimmunoprecipitation using monoclonal antibodies against NSP7α and NSP7β (Li et al., 2012).

Here, by Western blot using polyclonal antibodies against NSP7α and NSP7β, we found that cleaved NSP7α could be detected at 16 hpi, but NSP7 and NSP7β were still undetectable in the cell lysates infected by PRRSV. Recombinant NSP7β had been observed to be sensitive to degradation (Li et al., 2012). We speculated that the cleaved NSP7β could be rapidly degraded in virus infected cells, which resulted in the failures in the detection of the protein. For NSP7, however, we found in our studies that it could be abundantly expressed in *E. coli* and the purified protein was also quite stable (data not shown). Taken together with the fact that both antisera against NSP7α and NSP7β detected no NSP7 but some larger processing intermediates such as NSP3-8 and NSP5-7, our results indicated that PRRSV NSP7 might only exist as a short-lived intermediate if it was present. NSP7α was found stably existing as a cleavage product in PRRSV infected cells after 16 hpi, suggesting that it may play an important role in the virus infection. Whether the long-lived intermediates such as NSP3-8 and NSP5-7 act as functional proteins remains to be investigated.

It is noteworthy that PRRSV NSP7α contains a predominant PRR at the C-terminus. Its homologous region containing less proline residues presents at the N-terminus of EAV NSP7β, probably due to the cleavage site shift between NSP7α and NSP7β. PRRs widely exist in proteins and often have important functions. The proline-rich peptides are found to be especially involved in protein–protein interactions, such as the recognition of regulatory proteins with SH3 domains in signaling pathways. It has been found that the PRR in proteins with repetitive (XP)_n_ sequences can function as a stiff ‘sticky arm,’ binding rapidly and reversibly to other proteins (Williamson, 1994). The C-terminal PRR of PRRSV NSP7α has a sequence pattern of (XP)_8_ (**Figure 4**). It is a pity that we were unable to resolve the structure of this region due to the restriction of the NMR method we used in this study. Further studies are needed to understand whether this extra motif endows PRRSV NSP7α with any extra function than EAV NSP7α.

Structure analysis and comparison show that PRRSV NSP7α (PDB accession No. 5I65) and EAV NSP7α (PDB accession No. 2L8K) possess similar secondary structure elements (**Figure 5**). Both of them have three α-helices gathered together, and β-sheets of the two proteins are all located at one side of the α-helices. Helix α2 to helix α3 is the best superimposed region of two proteins (**Figure 5C**).

By yeast two hybrid and pull-down assays, we found that PRRSV NSP7α could interact with its viral RdRp. Site-directed mutagenesis analysis showed that NSP7α single mutants L69A and F72A lost the binding ability to NSP9. Interestingly, a previous study showed that a single mutation at F72 resulted in the failure in virus recovery by reverse genetics (Zhang et al., 2013). F72 is a conserved amino acid residue in NSP7α of PRRSV and EAV, indicating that it is probably a crucial residue for the protein to maintain its functional structure. Previous studies by reverse genetics have demonstrated that arterivirus NSP7α plays an important role in viral RNA synthesis (Manolaridis et al., 2011; Zhang et al., 2013). NSP9 acts as the viral RNA-dependent RNA polymerase and is a key component of the viral RTC (Knoops et al., 2012). The interaction of NSP7α with NSP9 imply that NSP7α might play a role in RTC and/or assist NSP9 to carry out its function in PRRSV RNA synthesis. Further work is needed to investigate whether and how the NSP7α–NSP9 interaction is involved in the virus replication.

To conclude, in this study, we investigated the expression of NSP7α and NSP7β in PRRSV-infected cells, resolved the structure of NSP7α in aqueous solution, and identified the interaction of NSP7α with the viral RNA dependent RNA polymerase NSP9. These data provided us some new insights into the molecular mechanism of PRRSV infectious cycle.

## Author Contributions

HC, XX, and JC designed the research; JC, XX, HT, and YW determined the solution structure of NSP7α; YL, HN, and MT demonstrated the interaction of NSP7α with NSP9; JC performed the rest of the experiments; JC, HC, and XX contributed to the writing of manuscript.

## Conflict of Interest Statement

The authors declare that the research was conducted in the absence of any commercial or financial relationships that could be construed as a potential conflict of interest. The reviewer TO and handling Editor declared their shared affiliation, and the handling Editor states that the process nevertheless met the standards of a fair and objective review.

## Footnotes

1 http://www.ebi.ac.uk/pdbe-site/pdbemotif/

2 http://cosbi.ku.edu.tr/prism/

3 http://www.nmr.ch

4 http://www.pymol.org/

5 http://www.rcsb.org/pdb/

6 http://www.ebi.ac.uk/pdbe-site/pdbemotif/
